# Peripersonal space plasticity, Self-disorders and intersubjectivity in patients with early-onset and adult-onset schizophrenia

**DOI:** 10.1192/j.eurpsy.2023.1050

**Published:** 2023-07-19

**Authors:** V. Lucarini, F. Magnani, F. Giustozzi, R. Volpe, F. Ferroni, M. Ardizzi, N. Fascendini, S. Amorosi, F. Rasmi, C. Marchesi, V. Gallese, M. Tonna

**Affiliations:** 1 Institute of Psychiatry and Neuroscience of Paris (IPNP), INSERM U1266, Université Paris Cité; 2 C’JAAD, Evaluation, Prevention and Therapeutic Innovation Department, GHU Paris Psychiatrie et Neurosciences; 3 Institut de Psychiatrie, CNRS GDR 3557, Paris, France; 4 Psychiatry Unit; 5 Unit of Neuroscience, University of Parma, Parma, Italy

## Abstract

**Introduction:**

In schizophrenia, there is evidence for anomalies in the extension and plasticity of the peripersonal space (PPS), the portion of space surrounding our body, plastically shaped through motor experiences. An impaired multisensory integration at the PPS level would underpin the disembodiment, a core feature of the disorder linked to subjective perturbations of the sense of self (“Self-disorders”) and of the intersubjective dimension (“schizophrenic autism”).

**Objectives:**

The present study was aimed at: 1) exploring possible associations between PPS data, psychopathological dimensions, and subjective experiences in schizophrenia; 2) identifying a specific PPS profile in patients with early-onset schizophrenia.

**Methods:**

A motor training with a tool was used to assess the PPS size and boundaries demarcation in twenty-seven schizophrenia outpatients. Moreover, they underwent a thorough psychopathological evaluation with the Positive And Negative Syndrome Scale (PANSS), the Examination of Anomalous Self Experience scale (EASE) and the Autism Rating Scale (ARS). Subsequently, the sample was divided into early (EOS) and adult-onset (AOS) subgroups, that were compared with respect to their PPS and psychopathological profiles.

**Results:**

PPS features (size and boundaries demarcation) were associated with PANSS negative score, subjective experiences of existential reorientation (EASE Domain 5 scores) and traits of schizophrenic autism (ARS scores; Fig. 1). PPS parameters (Fig. 2) and ARS scores, but not PANSS and EASE differentiated between early and adult-onset subgroups.

**Image:**

**Figure:**
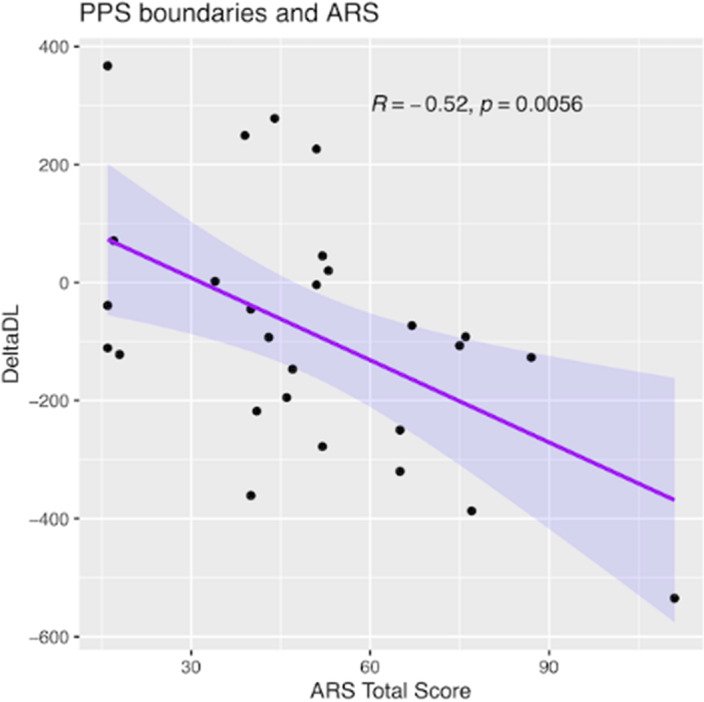

**Image 2:**

**Figure:**
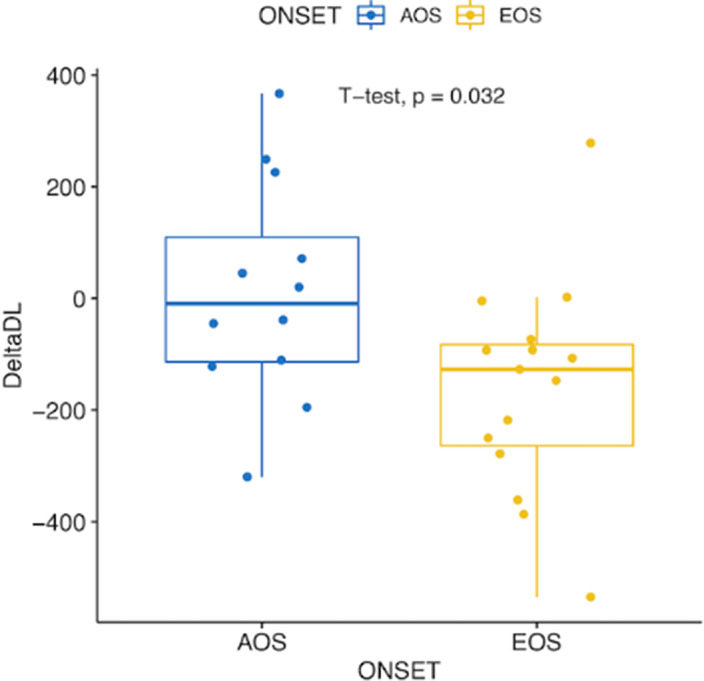

**Conclusions:**

Our results suggest a link between PPS patterns, negative symptoms, and disturbances of the subjective experience, particularly in the intersubjective domain, in schizophrenia. Moreover, they candidate specific PPS profiles and schizophrenic autism traits as EOS markers.

**Disclosure of Interest:**

None Declared

